# Communication strategies of the Cañariaco project and the environmental perception of citizens in a district of the Ferreñafe province, Perú

**DOI:** 10.12688/f1000research.167797.1

**Published:** 2025-12-01

**Authors:** Jordano Oneil Arcila Silva, Jorge José Novoa Cajusol, Gustavo Adolfo Ventura Seclén

**Affiliations:** 1 Universidad Cesar Vallejo, Trujillo, La Libertad, Peru

**Keywords:** Communication strategies, environmental perception, message clarity, communication channels, feedback.

## Abstract

**Background:**

This research supports SDG 17, partnerships for goals, as it will focus on the relationship between the Cañariaco Project’s communication strategies and citizens’ environmental perceptions, promoting transparency, informed participation, and institutional trust at the local level. The study aims to determine the relationship between the communication strategies of the Cañariaco project and the environmental perception of citizens in a district of the province of Ferreñafe 2025, Peru.

**Methods:**

The chosen methodology is quantitative, with a non-experimental, cross-sectional correlational design. The population consisted of 183 citizens, who were sampled using a representative sample of 125 participants using physical surveys composed of two previously validated instruments that assess the relevant constructs.

**Results:**

The analysis revealed that, while people show a high level of environmental awareness, the outreach tactics of the Cañariaco project are generally not considered very impactful. We noted that a significant portion of the survey participants felt that these tactics are at a low or medium level, which highlights certain deficiencies in the clarity of the message, the dissemination channels, and the feedback received. However, it was also discovered that there is a group with strong environmental awareness, which indicates concern and understanding about environmental issues, beyond the communication initiatives of the project. Additionally, the collected data diverges from studies that propose a direct connection between outreach tactics and environmental awareness, suggesting that other aspects may be influencing how the community perceives things.

**Conclusions:**

Consequently, it was concluded that the communication strategies of the Cañariaco project do not directly affect how the population perceives the environment. In reality, this perception is mainly built upon past experiences and the cultural values of the people. This shows us that simply improving external communication is not enough to foster trust and community participation.

## Introduction

When we refer to social problems related to mining around the world, we have the perfect example of one of the most controversial mining projects in Latin America, which was a contentious mining contract that has triggered the most significant protests and strikes in Panama in decades due to the large reserves of copper and gold. Subinas (2023) suggests that at the heart of this social unrest lies one of the largest copper reserves in the world, which, despite the social and environmental responsibilities of the companies involved, has not reached a complete agreement with the vulnerable populations, resulting in various active conflicts with these communities.

Another emblematic example is the Cerro Blanco project, located in Asunción Mita (Guatemala). Due to the various difficulties that arose, a different method of exploitation was chosen, switching from underground to surface mining. For this reason, in 2024, the Environmental Impact Study (EIA) was updated to ensure the project’s continuation. In this way, the company in charge of the project sought to engage with the community to inform them about the changes that would be made; in response, the community completely opposed mining. Unfortunately, Coyoy (2024) mentions that shortly after the results were presented, the Ministry of Energy and Mines (MEM) invalidated the consultation, proceeding with the process. This demonstrates a lack of understanding between the community, the authorities, and the company, concluding that the communication strategies implemented are ineffective and that the decision-making capacity of the community is being undermined.

If we look at a national case, we have the perfect example of the oldest social conflict in Arequipa, which is Tía María. The residents of the Tambo Valley do not accept the extraction of native copper related to the mining project of Southern Peru due to fears of possible environmental damage and impacts on agriculture. Zenaida (2021) mentioned that the socio-economic acceptance of mining is a topic where the population tends to be difficult to convince, which is why it is important to be consistent and visible with the communities near such potential projects.

Another mining project that has generated one of the most significant socio-environmental conflicts in the last decade is Conga, in Cajamarca. The rejection of the project led by the mining company Yanacocha was based on the severe impact on water, a fundamental resource for human consumption and agriculture. Protests spread throughout the country until the government suspended the project. Ormachea I (2022) points out that the situation becomes complicated when there are no equitable benefits between mining companies and communities, highlighting the urgency to improve communication techniques between the two.

Delving into the local level, in the province of Ferreñafe, more specifically in the district of Cañaris, we find the case of the Cañariaco project, which is a representative example of how the implementation of poor communication strategies can affect the course of a mining project. As a result, the community understands that the actions taken by the company to communicate about it are not assertive, generating a negative environmental perception, leading the community to internalize a distrust regarding the possible environmental effects that the exploitation of the deposit could cause. Some of the effects considered likely include water contamination, deforestation, and the modification or alteration of local ecosystems. This results in conflicts between the community, local authorities, and the project development company, thereby threatening the social viability of the project and restricting the community’s decision-making capacity regarding their environment and future.

To further illustrate the local level, we can talk about the Algarrobo project, located in Tambogrande, which has become one of the most controversial cases, encompassing social, economic, and environmental issues that are disputed among local residents. They firmly refuse to cede agricultural lands that will be granted by the state to the company in charge of the project; thus, the community accuses the state of prioritizing private interests over the well-being of the communities. Gonzales (2025) mentions that agriculture is the most important sector for national development; therefore, the exploitation of one of the largest copper reserves can generate significant controversy, as it directly affects the main source of income for local families. The study topic is linked to Sustainable Development Goal No. 16, which aims to promote equitable, peaceful, and inclusive societies. Within the context of this study, the problem has been proposed: What is the relationship between the communication strategies of the Cañariaco project and the environmental perception of citizens in a district of the Ferreñafe province in 2025?

This research work finds its justification, first from a theoretical angle, as it is based on the work of Santos Rodríguez (2023), who examines how ethical-environmental relationships in the mining sector are closely linked to the way individuals perceive their environment and extraction projects. From a practical perspective, the research is relevant as it aims to provide important data regarding the feelings of residents in the district in relation to the information they receive about the Cañariaco project. Finally, it is justified methodologically by implementing quantitative techniques (questionnaires).

Consequently, the general objective was set as: To determine the relationship between the communication strategies of the Cañariaco project and the environmental perception of citizens in a district of the Ferreñafe province in 2025. Additionally, the specific objectives were proposed as follows: To determine the level of implementation and effectiveness of the communication strategies used by the Cañariaco project in a district of the Ferreñafe province. Also, to assess the level of environmental perception of the citizens in the district regarding the development of the Cañariaco project. Lastly, to recognize the relationship between the dimensions of the communication strategies of the Cañariaco project and the environmental perception of citizens in a district of the Ferreñafe province in 2025.

### Theoretical framework

Quezada (2025) aimed to determine the understanding of the group involved in mining disputes. A methodology based on a cross-sectional descriptive design and a quantitative approach was used. The results indicated that the involved population has insecurity towards the company in charge of the project; this is evidenced by the fact that 70% of the communities involved are dissatisfied with the transparency of decision-making.

In line with this, Castillo et al. (2024) aimed to understand the perception of the involved groups regarding human impacts on a sanctuary. A descriptive methodology with a cross-sectional design was employed. It was found that all respondents value the sanctuary; a notable finding is that a large percentage (71.4%, p < 0.05) believes that the geological formations add significant interest to the sanctuary.

Similarly, Ávila et al. (2021) aimed to explore the environmental perception of pollution in the El Riito stream. A mixed methodology was implemented, analyzing both quantitative and qualitative information. The results showed that the population has a perception that the river pollution is high and very high, with 48% and 44%, respectively. Additionally, 77.40% are willing to participate in a program to reduce pollution.

In the same way, Aduvire (2023) aimed to issue initiation and completion certifications. To this end, a form was implemented to allow for the monitoring and incorporation of environmental impacts in areas occupied by mining, with the goal of achieving sustainable activity while considering the relationship with neighboring communities, eliminating risks of accidents and other potential sources of pollution. Once the results from this form are obtained, the Environmental Impact Study (EIA) will be considered, which serves to establish environmental conditions.

In turn, Flores et al. (2022) aimed to determine the relationship between the communication strategies implemented by the mining company and the perceptions of the residents in the direct influence area of the Tía María mining project regarding the social conflict during the period from 2009 to 2019. A quantitative methodology was employed, using questionnaires as the main instrument. The results showed a low perception of communication with the community.

Similarly, Hipolito et al. (2022) aimed to establish the connection between responsibility and environmental dialogue regarding solid waste resources (SWR) in the mining population of Ccochaccasa. A quantitative correlational descriptive methodology was applied. The result showed a p-value (4.68) far from the critical area (t = 1.652), indicating a correlation between responsibility and environmental communication.

Similarly, Flores (2021) aimed to establish the connection between the communication tactics implemented by the mining entity and the perspectives of the citizens in the immediate impact area of the mining process in Tía María. A non-experimental longitudinal quantitative methodology at a correlational level was employed. The results of the questionnaire were verified by a Cronbach’s alpha coefficient of 0.8272, with a total of 369 residents surveyed.

On the other hand, the following hypothesis was proposed: There is a relationship between the communication strategies of the Cañariaco project and the environmental perception of citizens in a district of the Ferreñafe province in the year 2025.

### Communication strategies

Definition. It is defined according to Ríos et al. (2020) as those that can facilitate the development and linguistic strengthening of different populations; however, certain terminologies within the strategies may hinder linguistic dialect. Færch (1984) also mentions that communication strategies are conscious plans that an individual uses to solve problems that make it difficult to achieve a communicative goal.

### Dimensions

The dimensions that encompass communication strategies are:

- Message clarity
- Communication channels
- Feedback

Within our research, we will consider the aforementioned dimensions, as we believe they are necessary for the application of our instrument.

### Environmental perception

Definition. According to Hildegardo’s (2002) criteria, it is conceptualized as the way an individual detects the surrounding environment, leading them to interact with it. Additionally, Nagar (2006) adds that various factors inherent to the environment and the qualities of the individual are involved, making it a process of familiarizing oneself with the environment through sensory information.

### Dimensions

Three crucial aspects are highlighted to determine the dimensions based on environmental perception:

- Environmental knowledge
- Attitudes toward the environment
- Perceived environmental responsibility

## Methodology

### Type, design, and scope

This research employed a basic type of investigation. According to Tamayo et al. (2003), basic research, also known as pure or fundamental research, occurs within a theoretical framework with the main objective of expanding knowledge and proposing theories. It precisely uses techniques such as sampling, so that its results can be applied to more complex contexts than those directly studied. It does not focus on the practical application of its results, as it considers that task to belong to other actors, not the researcher, being fundamental for building theories and concepts that explain social phenomena, such as citizens’ environmental perception regarding mining projects.

The research approach of this article was quantitative. According to Hernández et al. (2014), the approach is employed as a set of sequential and systematic processes, where each phase must follow the previous one, without the possibility of skipping any or advancing. Although some phases may be adjusted, the order is maintained without modification. The process begins with an idea that is defined, objectives and questions are established, the literature is reviewed, and a theoretical framework is created. Then, hypotheses are formulated, their testing is planned, and data is collected and analyzed to draw conclusions.

Regarding the design, it was non-experimental. According to Hernández et al. (2014), in this type of design, no variable is manipulated. In other words, the independent variable is not altered to assess its impact on other variables. Additionally, it is cross-sectional. In this sense, Hernández et al. (2014) notes that data is collected at a single point in time, with the purpose of detailing and evaluating the impact and interdependence at a specific moment.

To determine if the research has correlation, it is important to distinguish between the two variables that determine the variation among the factors. It is worth emphasizing that the main characteristic of correlational research is the straightforward handling of multiple variables simultaneously. As stated by Tamayo (2012), it is important to keep in mind that this covariation does not imply that there are causal relationships between the values.

### Population and sample

The population for the research. According to Ñaupas et al. (2018), it is defined as the total set of study elements that possess the required aspects. Additionally, it mentions that these elements can be biological or inert. In the present study, the population consisted of 183 people. To select the aforementioned population, the inclusion criteria applied were: individuals aged 18 or older, residing in the populated center of Cañaris, who have heard or received information about the Cañariaco project, and who have given their consent to participate voluntarily and knowingly. Regarding the exclusion criteria, the following was considered: illiterate individuals and/or those with disabilities that prevent them from responding to the questionnaire. The sample, according to Gómez (2006), is a significant part of the study elements or population. The sample in this study consisted of 125 people; this sample was selected through probabilistic sampling.

### Procedure

The procedure for conducting this study is divided into several key stages that allow us to understand the phenomenon in question within its context. It begins with a general idea, followed by the formulation of the problem to be addressed. Then, we design the methodology, select the appropriate samples, collect and process the information, and finally, interpret and analyze the results obtained to draw meaningful conclusions. the electronic consent of the participants was obtained.

### Techniques and instruments for data collection

Methods and tools for data collection are important as outlined by Hernández et al. (2014) as they span from in-depth interviews to structured surveys, direct observation, and more.

For this particular study, the survey was chosen as the primary method for information collection. As noted by Hernández et al. (2014), this technique centered on asking questions to a particular group with the aim of gathering pertinent information on a specific subject. This approach was selected because it enabled the formulation of clearly stated questions that aided in problem analysis and provided critical information for the study.

As a means of obtaining information in greater detail, a questionnaire was conducted. In the words of Hernández et al. (2014), “this tool enables the researcher to design a particular set of queries to extract very specific information” from a given population sample. For this particular study, the questionnaire was structured to include 30 questions organized into three dimensions for each variable. It was administered to the sample that had already been determined in order to obtain results that validated the stated hypothesis. Responses were measured using a Likert scale from 1 to 5, with “strongly disagree” mapped to a 1 and “strongly agree” a 5.

Regarding the observation about the evaluation tool used, it is stated that the research instrument is of original authorship. Therefore, obtaining a third-party copyright license is not applicable.

### Validity and reliability

In this scientific article, the survey was used as a technique and as an instrument 2 questionnaires were applied, which were validated by 3 experts in the field, these experts were Mg. Carla Mena Nevado, Mg. Helen Alberca Guerrero and Mg. Javier Salazar Ipanaque, developing its reliability through Cronbach’s alpha. In which for the first questionnaire it was 0.935 and in the second of 0.856.

Regarding the treatment of the data, descriptive statistics were used, since the levels of each variable were found evidenced in the first two specific objectives. Inferential statistics were also used, since the correlations between the variables and dimensions were found, everything was determined by processing the data in excel and SPSS version 26.

### Data analysis

To carry out the analysis of the information, it was first necessary to convert the obtained data into manageable formats that facilitated its interpretation. The study focused on understanding the environmental perception of citizens in relation to the communication strategies implemented in the Cañariaco Project, within a district of the Ferreñafe province. To this end, clear and precise objectives were defined, which allowed for a systematic and effective procedure, ensuring that the conclusions were well-founded and useful for the study.

To collect the information, a sample of 125 inhabitants from Cañaris was surveyed. During this process, each obtained data point was carefully reviewed to detect possible errors and ensure the accuracy of the results. This step was crucial to ensure the reliability of the information and to obtain a solid analysis of the identified problem.

The questionnaire used was based on the Likert scale, which allowed for a structured measurement of the respondents’ perceptions. Once the responses were collected through a digital form, the data were organized in Excel and numerically coded. Then, they were processed using IBM SPSS Statistics V.26, employing Cronbach’s alpha coefficient to assess the reliability of the information. Finally, the results were formatted according to APA guidelines, ensuring that each statistical analysis was correctly interpreted and could provide valuable information for the study.

### Ethical criterial

The research involved 125 individuals selected from the target population. The process began by sending each individual an email requesting their informed consent, which was returned in writing by the same email. Each participant was also provided with a detailed explanation of the study’s purpose, goals, and methodology. Data collection was carried out using digital tools such as virtual meetings via Zoom and surveys conducted using Google Forms, ensuring the confidentiality of the responses collected at all times.

Prior to their participation, all participants provided their informed consent. They were provided with a document describing the study’s purposes, the procedures contemplated, the potential benefits, possible risks, and confidentiality guarantees. Each participant signed this form before completing the questionnaire.

The ethics committee determined that no waiver of informed consent was appropriate, since the methodology used involved obtaining data through a questionnaire, making it essential that each participant explicitly and formally express their agreement to participate in the study.

Participants were also assured of their right to withdraw from the research at any time and without consequence, and the confidentiality of the information provided was guaranteed. To this end, codes were used instead of names, eliminating any possibility of personally identifying the respondents.

The research does not involve medical interventions or experimental procedures that could compromise the safety or well-being of the participants. Likewise, compliance with the standards for the protection and care of the subjects involved will be guaranteed, following the guidelines established by the institution and current national and international legislation.

## Results

### Description of the results of the first variable: Strategic communication

The results obtained regarding the variable *Strategic Communication*, presented in Table 1, show that 48.8% of respondents perceive a low level in the application of communication strategies, while 42.4% consider the level to be medium, and only 8.8% perceive it as high. This indicates that most of the evaluated population perceives deficiencies in the implemented communication strategies, which may reflect a lack of effective mechanisms for conveying environmental messages.

**Table 1.** Levels of communication strategies.

		Frequency	Percentage
Valid	Low	61	48,8
	Medium	53	42,4
	High	11	8,8
	Total	125	100,0

### Description of the results of the second variable: Environmental perception

Regarding the variable Environmental Perception, the results presented in Table 2 show that 60% of the participants demonstrate a high level of perception, 37.6% a medium level, and only 2.4% a low level. These results reflect favorable knowledge and awareness toward the environment, although there are still groups with intermediate perceptions that require educational reinforcement.

**Table 2.** Levels of environmental perception.

		Frequency	Percentage
Valid	Low	3	2.4
	Medium	47	37,6
	High	75	60,0
	Total	125	100,0

### Correlation between the dimensions of strategic communication and environmental perception

To determine the relationship between the dimensions of strategic communication and environmental perception, the Spearman correlation test was applied. The results indicate that none of the dimensions (message clarity, communication channels, and feedback) showed a significant relationship with environmental perception, since the bilateral significance values were greater than 0.05, as shown in Tables 3, 4, and 5.

**Table 3.** Relationship of dimension 1 of communication strategies with environmental perception.

Correlations		Message clarity	Environmental perception
Message clarity	Correlation coefficient	1	,107
	Sig. (bilateral)	.	,234
	N°	125	125
Environmental perception	Correlation coefficient	,107	1
	Sig. (bilateral)	,234	.
	N°	125	125

**Table 4.** Relationship of dimension 2 of communication strategies with environmental perception.

Correlations		Communication channels	Environmental perception
Communication channels	Correlation coefficient	1	-,004
	Sig. (bilateral)	.	,967
	N°	125	125
Environmental perception	Correlation coefficient	-,004	1
	Sig. (bilateral)	,967	.
	N°	125	125

**Table 5.** Relationship of dimension 3 of communication strategies with environmental perception.

Correlations		Feedback	Environmental perception
Feedback	Correlation coefficient	1	,046
	Sig. (bilateral)	.	,608
	N°	125	125
Environmental perception	Correlation coefficient	,046	1
	Sig. (bilateral)	,608	.
	N°	125	125

Specifically, message clarity obtained a *p*-value of 0.234, communication channels *p* = 0.967, and feedback *p* = 0.608, which demonstrates the absence of a statistically significant correlation. This suggests that the current communication strategies are not directly influencing the population’s level of environmental perception.

### General correlation between strategic communication and environmental perception

Finally, the overall relationship between both variables was analyzed, obtaining a correlation coefficient of 0.027 with a bilateral significance of 0.765, as detailed in Table 6. This result confirms that there is no statistically significant relationship between strategic communication and environmental perception in the population of Cañaris. It is inferred that institutional or community communication may lack relevance and effective strategies to strengthen environmental awareness.

**Table 6.** Relationship between communication strategies and environmental perception.

Correlations		Communication strategies	Environmental perception
Communication strategies	Correlation coefficient	1	,027
	Sig. (bilateral)	.	,765
	N°	125	125
Environmental perception	Correlation coefficient	,027	1
	Sig. (bilateral)	,765	.
	N°	125	125

### Reliability of the instruments

The results of the reliability analysis using Cronbach’s alpha coefficient showed appropriate values for both variables. The variable *Strategic Communication* obtained α = 0.935, and the variable *Environmental Perception* obtained α = 0.856. These values confirm the internal consistency and reliability of the instruments used.

### Normality tests

According to the Kolmogorov–Smirnov test, none of the variables follow a normal distribution (p < 0.05), with values of 0.005 for *Strategic Communication* and 0.000 for *Environmental Perception.* For this reason, non-parametric tests were used in the correlational analysis.

## Discussion - Conclusions - Recommendations

Based on the general objective, it was concluded that there is no association between communication strategies and the environmental perception of the citizen regarding the Cañariaco project. This result is due to the fact that, although communication strategies have deficiencies and limitations, the environmental perception of the community is determined, in the first place, by sufficient internal factors such as past experiences, cultural values and deep-rooted beliefs. Consequently, external communication cannot become the only determining factor. The final recommendation would be that interventions should not only be aimed at improving communication strategies, but also at some processes of participatory dialogue and even environmental education that addresses the beliefs and values of the community, which would promote a more mature and contextualized understanding for a more constructive coexistence of the population and the project.

Likewise, with regard to specific objective 01: it was determined that the majority of the population perceives deficiencies or that the communication strategies implemented are insufficient. Reflecting a negative assessment, while a small minority consider them to be adequate and positive. In this sense, the perception of the population reveals a disconnection between the communication offered and the expectations or information needs of the community. This may affect community trust and participation related to the Cañariaco project. Therefore, it is recommended to develop and reformulate the communication strategies used, directing them towards greater clarity, inclusion and cultural relevance. That allows them to improve interaction with the directly affected population, creating the conditions for a more positive and constructive general perception.

In the same way, in relation to specific objective 2: it was concluded that the majority of the population evaluated has a high environmental perception, while only a minority proportion maintains a medium perception and practically a minimum part has a low perception. This indicates that citizens in general are informed and value the importance of the environment and consider its care relevant, although the perception is not directly related to the communication strategies applied around the Cañariaco project. Therefore, it is suggested to use the existing knowledge about the environment for the creation of more effective and contextualized communication strategies, which seek to promote a stronger citizen participation and collaborative management of the environment, promoting the sustainability of the project.

Finally, with respect to specific objective 3: In this way, it can be deduced that there is no relationship between the dimensions of the communication strategies variable and the environmental perception of the citizen regarding the Cañariaco project, since none of the components of communication has a direct impact on how the community appreciates and perceives its environment. This circumstance indicates that, despite the shortcomings in the messages transmitted or in the feedback systems, the perception of the environment arises from a series of deeper, more complex and determining dimensions. These may be related to previous experiences, cultural principles and convictions deeply rooted in the community. Therefore, it is advisable that communication tactics be enriched with educational and participatory processes that deal with these internal dimensions, encouraging a more intimate and contextual exchange. This will facilitate a better understanding and assessment of the environment, as well as a more constructive relationship between the community and the project.

## Ethics and consent

The study included 125 participants from the given population. Participation began with an in-person survey, which requested their informed consent, which was received in writing via the same means. Participants were also provided with detailed explanations about the study, including its objectives and procedures.

The research was conducted in accordance with the ethical principles established in the Declaration of Helsinki, which protects individuals involved in research. Each participant was given clear instructions and explanations about the study’s objectives, and all were offered the option to withdraw from participation at any time without repercussions. Personal information was kept confidential during and after the completion of the research.

All participants received informed consent before the start of their participation. Each participant was provided with a document detailing the study’s objectives, procedures, benefits, risks, and potential confidentiality concerns. They signed this document before completing the questionnaire. The study was conducted in compliance with the approval granted by the Institutional Ethics Committee of César Vallejo University, under code 659-2024, and adheres to the ethical principles of the Declaration of Helsinki and relevant national legislation.

The ethics committee did not grant any exemptions for the consent process, as the study methodology involved data collection through a questionnaire. It was essential that all participants provide explicit and formal consent.

Furthermore, participants were assured of confidentiality that protected their identities and provided them with the right to withdraw their participation at any time without facing negative consequences. Risks associated with personal and sensitive data were mitigated through the use of coded responses.

### Reporting guidelines

Zenodo. STROBE checklist for Communication strategies of the Cañariaco project and the environmental perception of citizens in a district of the Ferreñafe province, Perú https://doi.org/10.5281/zenodo.17315826 ( Arcila et al., 2025).

The data are available under the terms of the Creative Commons Attribution 4.0 International license (CC-BY 4.0).

## Data Availability

Zenodo: Communication strategies of the Cañariaco project and the environmental perception of citizens in a district of the Ferreñafe province, Perú https://doi.org/10.5281/zenodo.17315826 ( Arcila et al., 2025). This article contains the following underlying data:
•
Communication strategies.xlsx
•
Environmental perception.xlsx
•
RESEARCH METHODOLOGY.xlsx The data are available under the terms of the Creative Commons Attribution 4.0 International license (CC-BY 4.0). Zenodo: Communication strategies of the Cañariaco project and the environmental perception of citizens in a district of the Ferreñafe province, Perú https://doi.org/10.5281/zenodo.17315826 ( Arcila et al., 2025). This project includes the following extended data: Informed consent The data are available under the terms of the Creative Commons Attribution 4.0 International license (CC-BY 4.0).
