# Communication strategies of the Cañariaco Project and the Environmental Perception of Citizens in a District of the Ferreñafe Province, Perú

**DOI:** 10.12688/f1000research.167797.2

**Published:** 2026-08-08

**Authors:** Jordano Oneil Arcila Silva, Jorge José Novoa Cajusol, Gustavo Adolfo Ventura Seclén

**Affiliations:** 1 Universidad Cesar Vallejo, Trujillo, La Libertad, Peru

**Keywords:** Communication strategies, environmental perception, message clarity, communication channels, feedback.

## Abstract

**Background:**

This study aimed to determine the relationship between the communication strategies of the Cañariaco project and the environmental perception of citizens in a district of the Ferreñafe province in 2025.

**Methods:**

A quantitative, non-experimental, cross-sectional, and analytical-correlational study was conducted. From a population of 183 citizens, a sample of 125 participants was calculated, with a 95% confidence level and a 5% margin of error. Recruitment was conducted in person, consecutively, and based on availability. Two 15-item questionnaires with a five-point Likert scale were administered. The analysis included descriptive statistics, Cronbach’s alpha, Spearman’s rank correlation with bootstrap confidence intervals, multiple linear regression with robust HC3 errors, and sensitivity analysis.

**Results:**

The communication strategies obtained a mean score of 37.37 (SD = 14.23); 48.8% of respondents presented a low level of perception. Environmental perception reached a mean of 57.94 (SD = 9.19), with 60.0% at a high level. The overall assessment was practically null and not significant (rho = 0.027; p = 0.765; 95% CI: −0.161 to 0.207). No significant bivariate associations were found in the dimensions either. The regression model explained 6.7% of the variability and showed borderline overall significance (F = 2.61; p = 0.055). Only message clarity showed an adjusted positive association (b = 0.798; 95% CI: 0.224 to 1.373; p = 0.007).

**Conclusions:**

No evidence of association was found between the overall assessments of both variables. Message clarity could provide additional information, although the results do not allow us to establish causality. Probabilistic, longitudinal studies with sociodemographic variables and bilingual instruments are recommended.

## Introduction

Communication in extractive projects cannot be reduced to simply issuing messages or institutional promotion. In territories where water, agriculture, communal identity, and the continuity of livelihoods are perceived as threatened, corporate information is interpreted through pre-existing relationships of trust, experiences of participation, the legitimacy of the actors involved, and social judgments about risk. For this reason, strategic communication must be understood as a deliberate organizational process that links mission, decisions, audiences, channels, listening, and evaluation, not as a linguistic technique for overcoming difficulties in a second language ( Hallahan et al., 2007).

This approach is particularly relevant in mining. The social license to operate is not a legal authorization, but rather a dynamic level of community acceptance that depends on legitimacy, credibility, and trust ( Thomson & Boutilier, 2011). Peruvian evidence shows that these conditions are built through verifiable behaviors, fulfillment of commitments, recognition of local interests, and sustained relationships, and not solely through information campaigns ( Saenz, 2019). Complementarily, the psychometric paradigm of risk perception explains that public judgments about potentially dangerous technologies or activities incorporate dimensions such as fear, uncertainty, control, distribution of benefits, and trust in institutions ( Slovic, 1987).

Peruvian mining conflicts demonstrate that the perception of economic benefits can coexist with concerns about environmental impacts, territorial inequality, and inadequate service provision. An analysis by the Inter-American Development Bank identifies dissatisfaction with local development and insufficient access to basic services among the factors fueling conflict surrounding mining projects ( Bustamante Suárez, 2024). In this context, community communication can help clarify information and create spaces for feedback, but it does not replace meaningful participation, environmental performance, or accountability.

The district of Cañaris is located in the province of Ferreñafe, Lambayeque region. The San Juan de Cañaris Peasant Community maintains a territorial identity linked to the Quechua language Inkawasi-Cañaris, small-scale agriculture and livestock farming, and local water sources. The Cañariaco copper project was operated during the study period by Alta Copper Corp., formerly known as Candente Copper Corp. The Ombudsman’s Office registered the case as an active socio-environmental conflict since March 2012, associated with concerns about environmental impacts and demands for dialogue. In March 2026, Fortescue reported that it had completed the acquisition of Alta Copper; this subsequent change does not alter the corporate context under which the data were collected in 2025, but it should be noted to avoid historical ambiguity.

The research question was: what is the relationship between the communication strategies attributed to the Cañariaco project and the environmental perception of citizens surveyed in a district of the Ferreñafe province during 2025? The study aligns with SDG 16 because it examines conditions related to access to information, transparency, listening, trust, and informed participation. The intended contribution is empirical and delimited: to estimate the magnitude and precision of the observed associations, without asserting causality or attributing the results to factors not included in the questionnaire.

Consequently, the overall objective was defined as: To determine the relationship between the communication strategies of the Cañariaco project and the environmental perception of citizens in a district of the Ferreñafe province in 2025. In addition, the following specific objectives were proposed: To describe the level of the communication strategies, both in terms of overall score and message clarity, communication channels, and feedback. Likewise, to describe the level of environmental perception, considering environmental knowledge, attitudes toward the environment, and perceived environmental responsibility. Similarly, to estimate the bivariate association between each dimension of the communication strategies and environmental perception, with confidence intervals. Finally, to examine the simultaneous contribution of the three communication dimensions using a robust multiple regression model.

**Research Hypothesis**. Communication strategy scores are associated with environmental perception. For multiple analysis, it was exploratoryly assessed whether clarity, channels, and feedback provided independent information when modeling the total environmental perception score.

## Theoretical framework

### Strategic organizational communication

Definition. Hallahan et al. (2007) define strategic communication as the intentional use of communication by an organization to fulfill its mission. This definition integrates planning, execution, and evaluation, and requires considering not only what the organization wishes to convey, but also how its decisions are interpreted by audiences with different interests, knowledge, and power dynamics. In a mining project, a scientifically evaluable communication strategy must demonstrate coherence among messages, channels, information timeliness, and response mechanisms.

### Dimensions

In this study, the variable was operationalized using three dimensions already present in the original instrument:

• Message clarity
• Communication channels
• Feedback

Clarity refers to comprehensibility, precision, and consistency; channels represent accessibility and suitability of the media used; and feedback expresses the possibility of asking questions, obtaining answers, and recognizing that communication is bidirectional. These dimensions alone do not encompass all of strategic communication, so the results should be interpreted as a partial evaluation of the construct.

### Social license to operate, legitimacy, and trust

The social license to operate describes a continuously negotiated social acceptance. Thomson and Boutilier (2011) explain that it can progress from minimal acceptance to relationships based on trust and identification. Legitimacy is linked to the perception that activities are appropriate and respect shared norms; credibility requires congruence between words and actions; and trust implies a willingness to accept vulnerability to the behavior of another actor. In the Peruvian case, Saenz (2019) found that mining companies strengthen their social license when they articulate pragmatic, moral, and cognitive legitimacy with trust based on integrity and competence.

This framework allows us to avoid an instrumental interpretation according to which more intense communication should automatically produce environmental acceptance. A community can perfectly understand a message and, at the same time, disagree with the project. Therefore, a low correlation between communication and perception does not demonstrate the irrelevance of communication; This may indicate that different domains are being measured, that there is unobserved heterogeneity, or that the instrument does not capture dimensions such as trust, compliance, procedural fairness, or participation.

### Environmental risk perception

Risk perception refers to subjective judgments about the probability, severity, and acceptability of threats. Slovic (1987) showed that such judgments are not derived solely from technical information: familiarity, perceived control, fear, potential consequences, and trust all play a role. In socio-environmental conflicts, perception can intensify when benefits and risks are distributed unequally or when institutions are considered opaque. The present study did not directly measure perceived risk or institutional trust; these concepts are incorporated to theoretically define the phenomenon and guide subsequent research, not to causally explain the results obtained.

## Environmental perception

Definition. According to Córdova (2002) criteria, it is conceptualized as the way an individual detects the surrounding environment, leading them to interact with it. Additionally, Nagar (2006) adds that various factors inherent to the environment and the qualities of the individual are involved, making it a process of familiarizing oneself with the environment through sensory information.

### Dimensions

Environmental perception encompasses how people recognize, interpret, and value their environment. The instrument analyzed:

• Environmental knowledge
• Attitudes toward the environment
• Perceived environmental responsibility

Knowledge includes declared information or understanding; attitudes express valuation and a favorable disposition toward care; and perceived responsibility relates to the attribution of duties and protective actions. The total score summarizes these components, but does not equate to a direct measurement of mining risk, business confidence, or social license.

## Empirical Background

Studies on mining and communication in Peru indicate that the quality of the relationship with communities depends on transparency, participation, and territorial relevance. Flores Castro (2021) and Flores-Castro et al. (2022) analyzed communication surrounding the Tía María project and reported a limited assessment of communication processes. Vargas-Sacha et al. (2022) studied waste management and environmental communication in a mining community; in the previous version of this manuscript, “p = 4.68” was erroneously recorded. This notation was removed because a p-value ranges from 0 to 1; the number 4.68 should be treated, according to the original analysis, as a test statistic and not as a probability.

Other studies show that environmental perceptions can coexist with a willingness to participate in conservation programs and with critical impact assessments. González Ávila et al. (2021) found high perceived levels of pollution in an urban stream and a strong willingness to participate in its cleanup. Castillo Paredes et al. (2024) documented the assessment of natural and geological elements in a sanctuary. This background supports a multidimensional interpretation: environmental knowledge and attitudes are not simply products of a communication campaign, but rather the result of local experiences, available information, and frameworks of social valuation. In the present study, however, only the variables that were actually measured can be analyzed.

## Methodology

### Type, design, and scope

A quantitative, non-experimental, cross-sectional study with an analytical-correlational scope was conducted. Variables were observed without manipulation and at a single point in time. Inference is limited to statistical associations within the sample; the design does not establish temporal precedence or causality. The expansion to multiple regression aims to estimate adjusted associations between communication dimensions, not to demonstrate the effects of communication on environmental perception.

This research employed a basic type of investigation. According to Tamayo & Tamayo (2003), basic research, also known as pure or fundamental research, occurs within a theoretical framework with the main objective of expanding knowledge and proposing theories. It precisely uses techniques such as sampling, so that its results can be applied to more complex contexts than those directly studied. It does not focus on the practical application of its results, as it considers that task to belong to other actors, not the researcher, being fundamental for building theories and concepts that explain social phenomena, such as citizens’ environmental perception regarding mining projects.

The research approach of this article was quantitative. According to Hernández et al. (2014), the approach is employed as a set of sequential and systematic processes, where each phase must follow the previous one, without the possibility of skipping any or advancing. Although some phases may be adjusted, the order is maintained without modification. The process begins with an idea that is defined, objectives and questions are established, the literature is reviewed, and a theoretical framework is created. Then, hypotheses are formulated, their testing is planned, and data is collected and analyzed to draw conclusions.

Regarding the design, it was non-experimental. According to Hernández et al. (2014), in this type of design, no variable is manipulated. In other words, the independent variable is not altered to assess its impact on other variables. Additionally, it is cross-sectional. In this sense, Hernández et al. (2014) notes that data is collected at a single point in time, with the purpose of detailing and evaluating the impact and interdependence at a specific moment.

To determine if the research has correlation, it is important to distinguish between the two variables that determine the variation among the factors. It is worth emphasizing that the main characteristic of correlational research is the straightforward handling of multiple variables simultaneously. As stated by Tamayo (2012), it is important to keep in mind that this covariation does not imply that there are causal relationships between the values.

### Population and sample

The fieldwork was conducted during 2025 in the urban area of the Cañaris district, Ferreñafe province, Lambayeque. At that time, the Cañariaco project was under the control of Alta Copper Corp. The acquisition by Fortescue was completed on March 10, 2026, after the data collection; therefore, the items were answered with reference to the project and the communication perceived within the corporate context of 2025.

The reference population consisted of 183 adult citizens linked to the area delimited for the study. The required sample size was calculated using the formula for proportions in finite populations:

$$n=\left(N\times Z^{2}\times p\times q\right)/\left[e^{2}\times \left(N-1\right)+Z^{2}\times p\times q\right]$$

With N = 183, Z = 1.96, p = 0.50, q = 0.50, and e = 0.05, the result was n = 124.17, rounded to 125 participants. This formula justifies the required number, but it does not automatically make the actual procedure probabilistic. Since there is no complete selection list or evidence of random selection in the files, recruitment is transparently described as consecutive, based on availability, among eligible individuals encountered during fieldwork. Consequently, the estimates should not be generalized as if they were derived from a simple random sample. To select the aforementioned population, the inclusion criteria applied were: being 18 years of age or older, residing within the study area, and having prior knowledge of or exposure to information about the Cañariaco project, as well as voluntary acceptance and signing of the informed consent form.

No one was excluded due to illiteracy or disability. When necessary, the team conducted a guided reading, explained the alternatives without suggesting answers, and allowed the participant to indicate their choice. This adaptation reduces barriers, although any future replication should individually document the type of support used and apply formal accessibility standards.

### Procedure

Data collection was conducted exclusively in person using printed questionnaires. Researchers explained the purpose, the voluntary nature of participation, the confidentiality of data processing, and the right to withdraw. Written consent was obtained during the same meeting. Google Forms, Zoom, or email consent forms were not used. Responses were coded with identifiers E1 to E125, digitized in spreadsheets, and subsequently checked for missing or out-of-range values. The two databases provided contain 125 complete records and valid ratings (1 to 5) across the 30 articles.

### Administration language

According to the protocol described by the authors, the questionnaire and the explanation of the consent form were available in Spanish and Quechua (Inkawasi-Cañaris). Participants could request oral assistance in their preferred language. The analytical database does not record which version each participant used, nor does it include an indicator of their native language; therefore, it was not possible to compare responses by language, assess metric equivalence, or verify invariance between versions. Before a new request is made, the original texts of both versions, the cross-cultural translation and review process, and a field identifying the administration method should be included.

### Techniques and instruments for data collection

As shown in Table 1, the technique used was a survey, and two questionnaires developed by the team were employed, each with 15 items and a five-point Likert scale: 1 = strongly disagree, 2 = disagree, 3 = neither agree nor disagree, 4 = agree, and 5 = strongly agree. The instruments were reviewed by three experts: Carla Mena Nevado, Helen Alberca Guerrero, and Javier Salazar Ipanaqué. The previous version only reported overall reliability; in this revision, internal consistency was recalculated for each instrument and dimension.

**Table 1.** Structure, scoring and interpretation of the instruments.

Variable	Dimensions and Items	Range	Total Interpretation
Communication Strategies	Clarity: P1–P5; channels: P6–P10; feedback: P11–P15	15–75	Low: 15–35; medium: 36–56; high: 57–75
Environmental Perception	Knowledge: P1–P5; attitudes: P6–P10; responsibility: P11–P15	15–75	Low: 15–35; medium: 36–56; high: 57–75

For each five-item dimension, the equivalent descriptive ranges are: low = 5–11, medium = 12–18, and high = 19–25. Inferential analysis used continuous scores because categorization reduces information and statistical power. The literal text of the items is not included in the two provided databases; for scientific integrity, it must be incorporated from the original instrument as supplementary material, without being reconstructed or inferred from codes P1–P15.

### Sociodemographic variables

The provided databases do not include age, sex, education, occupation, native language, or length of residence. Consequently, it is not possible to describe the sociodemographic composition of the sample or control for confounding factors using these covariates. This omission is explicitly stated and acknowledged as a material limitation. If separate data sheets exist, they must be integrated using an anonymous identifier before application; if they do not exist, it is inappropriate to fabricate percentages or attribute demographic representativeness to the study group.

### Validity and reliability

In this scientific article, the survey was used as a technique and as an instrument 2 questionnaires were applied, which were validated by 3 experts in the field, these experts were Mg. Carla Mena Nevado, Mg. Helen Alberca Guerrero and Mg. Javier Salazar Ipanaque.

Internal consistency was estimated directly from the items using Cronbach’s alpha. For communication strategies, α = 0.937 was obtained, with adequate values for each dimension. For environmental perception, α = 0.838 was obtained; the environmental knowledge dimension showed α = 0.542, a value that warrants caution as it suggests item heterogeneity or low covariation. The attitudes and responsibility dimensions showed acceptable consistency. Reliability does not demonstrate construct validity; therefore, future versions should incorporate factor analysis and cross-cultural evaluation.

## Data analysis

• Data cleaning and verification of ranges, missing values, and consistency of sums. No missing data were identified in the analyzed variables.
• Descriptive statistics using frequency, percentage, mean, standard deviation, median, range, and interquartile range.
• Internal consistency using Cronbach’s alpha (overall and by dimension).
• Bivariate associations using Spearman’s rho. Precision was estimated using percentile confidence intervals obtained from 10,000 bootstrap resamples.
• Multiple linear regression with overall environmental perception as the outcome and clarity, channels, and feedback as simultaneous predictors. Due to non-normality of residuals and heteroscedasticity, the main inference used robust HC3 standard errors.
• Collinearity was assessed using variance inflation factors (VIF), residual normality using Shapiro-Wilk, and heteroscedasticity using Breusch-Pagan and White.
• Sensitivity analysis: minimum detectable correlation with 80% power and minimum detectable R ^2^ for three predictors. Post hoc power was not interpreted as proof of the null hypothesis.

A two-tailed α = 0.05 was adopted. P-values were interpreted in conjunction with association sizes, confidence intervals, and explained variance. The analyses were replicated using the two provided databases; the codebook and results were prepared in a separate file.

### Ethical criterial

Participation was voluntary, and written informed consent was obtained before administering the questionnaire. Objectives, procedures, foreseeable risks, confidentiality, and the right to withdraw without consequence were communicated. Records were anonymized using codes and used for academic purposes. The study was conducted in accordance with the César Vallejo University Research Ethics Code, version 02, approved by University Council Resolution RCPU No. 0659-2024-UCV, and underwent institutional review, as reported by the authors. The absence of direct identifiers reduces the risk of re-identification, although the complete omission of demographic variables limits the assessment of equity and representativeness.

## Results

### Database integrity and available features

One hundred and twenty-five questionnaires with 30 valid responses per participant were analyzed. No missing data were observed in the items, dimensions, or total scores. The available information allows for the description of the scales and the analysis of their relationships, but not the creation of a demographic table. Therefore, the results refer to “surveyed participants” and not to subgroups of age, sex, education, language, or occupation.

### Internal consistency

The overall Cronbach’s alpha for the environmental perception variable, recalculated from the items presented in Table 2, was 0.838, replacing the previously reported value of 0.856. This difference may be due to a different calculation method, rounding criteria, or the use of a different version of the database. For transparency and reproducibility, the manuscript reports the coefficient obtained directly from the data presented in Table 2 and from the deposited files.

**Table 2.** Internal consistency of the instruments and their dimensions.

Scale or dimension	Items	Cronbach's alpha	Methodological Interpretation
Total communication strategies	15	0,937	High internal consistency
Message clarity	5	0,813	Adequate
Communication channels	5	0,858	Adequate
Feedback	5	0,887	Adequate
Overall environmental perception	15	0,838	Adequate for overall analysis
Environmental knowledge	5	0,542	Low; interpret with caution
Attitudes toward the environment	5	0,738	Acceptable
Perceived environmental responsibility	5	0,795	Adequate

### Description of the results of the first variable: Strategic communication

According to the results presented in Tables 3 and 4, almost half of the participants rated the communication strategies as low, while only 8.8% rated them as high. Furthermore, the feedback dimension registered the lowest average among the dimensions evaluated. These results reflect an unfavorable assessment of the communication strategies as perceived by the respondents; however, they do not allow us to identify the causes of this assessment or to conclude that the company objectively implemented an ineffective communication strategy, since the variable evaluated the participants’ perceptions and not an objective measurement of the organization’s performance.

**Table 3.** Descriptive statistics of communication strategies.

Score	Mín.	Máx.	Mean	SD	Median	IQR
Total communication strategies	15	69	37,37	14,23	38	24
Message clarity	5	25	12,90	4,85	12	—
Communication channels	5	25	12,54	5,10	11	—
Feedback	5	23	11,92	5,58	11	—

**Table 4.** Level of communication strategies.

Level	Frequency	Percentage
Low	61	48,8%
Medium	53	42,4%
High	11	8,8%
Total	125	100,0%

### Description of the results of the second variable: Environmental perception

According to the results presented in Tables 5 and 6, the total environmental perception score was concentrated in the medium and high levels. This result indicates a favorable assessment of environmental perception within the applied instrument; however, it should not be automatically interpreted as evidence of sufficient technical knowledge, acceptance or rejection of the project, or a specific level of perceived risk, since these constructs represent conceptually distinct dimensions and were not directly measured by the instrument.

**Table 5.** Descriptive statistics of environmental perception.

Score	Mín.	Máx.	Mean	SD	Median	IQR
Overall Environmental Perception	27	73	57,94	9,19	61	13
Environmental Knowledge	7	24	17,20	3,46	—	—
Attitudes Toward the Environment	5	25	19,82	4,00	—	—
Perceived Environmental Responsibility	5	25	20,91	3,74	—	—

**Table 6.** Level of environmental perception.

Level	Frequency	Percentage
Low	3	2,4%
Medium	47	37,6%
High	75	60,0%
Total	125	100,0%

### Bivariate associations

According to the results presented in Table 7, all confidence intervals included zero. In particular, the overall correlation estimate was very close to zero, and its confidence interval was compatible with both a small negative and a small positive association. Consequently, the results indicate that the sample did not provide statistically significant evidence of a monotonic association between the variables evaluated. Therefore, it is not appropriate to state that the null hypothesis has been “accepted” or “proven,” nor to conclude that there is a definitive absence of a relationship in the population, but only that, with the available evidence, no statistically significant association was detected.

**Table 7.** Spearman correlations with total environmental perception.

Predictor	rho	p	IC95% bootstrap	Observed magnitude
Message clarity	0,107	0,234	-0,082 a 0,289	Very low
Communication channels	-0,004	0,967	-0,196 a 0,188	Practically zero
Feedback	0,046	0,608	-0,135 a 0,225	Practically zero
Total communication strategies	0,027	0,765	-0,161 a 0,207	Practically zero

### Multiple linear regression

According to the results presented in Table 8, a multiple regression model was estimated with overall environmental perception as the dependent variable and the three dimensions of communication strategies as simultaneous predictors. The variance inflation factors (VIF) were 2.68 for clarity, 3.40 for channels, and 2.86 for feedback, values that did not exceed the threshold of 5, although they showed a relatively high correlation among the predictor dimensions. Furthermore, the model assumptions showed that the residuals did not follow a normal distribution (Shapiro–Wilk, p = 0.004) and that heteroscedasticity existed, according to the Breusch–Pagan (p = 0.035) and White (p = 0.025) tests. Consequently, the model coefficients were reported using robust HC3 standard errors, which provides more reliable inferences in the face of these assumptions being violated.

The clarity coefficient indicates that, holding channels and feedback constant, one additional point in clarity was associated with 0.80 additional points in environmental perception. This result did not appear in the bivariate correlation and emerged when controlling for two highly related dimensions; therefore, it may reflect a conditional or suppression effect. The robust overall test was just above 0.05, and the explained variance was reduced. It should not be presented as causal evidence or as strong confirmation that increasing clarity will produce environmental changes.

**Table 8.** Robust multiple regression for total environmental perception.

Variable	b	EE HC3	β est.	t	p	IC95% de b
Constant	54,689	2,757	—	19,84	<0,001	49,230 a 60,148
Message clarity	0,798	0,290	0,421	2,75	0,007	0,224 a 1,373
Communication channels	-0,438	0,327	-0,243	-1,34	0,184	-1,086 a 0,210
Feedback	-0,131	0,286	-0,079	-0,46	0,649	-0,698 a 0,436

*Note*: n = 125. R ^2^ = 0.067; adjusted R ^2^ = 0.044. Robust overall test F(3, 121) = 2.61; p = 0.055.

### Sensibilidad estadística

According to the results presented in Table 9, the sample showed adequate sensitivity for detecting correlations of approximately 0.25 or higher, although it may not have had sufficient power to identify associations of smaller magnitude. Furthermore, the observed R ^2^ was lower than the approximate R ^2^ required to achieve a statistical power of 80%. Consequently, the overall lack of significance of the model can be attributed to the existence of a small effect size, the inherent variability of the sample, or the absence of an association between the variables. Therefore, with the available evidence, the results do not allow for a conclusive determination of which of these explanations is correct.

**Table 9.** Sensitivity analysis of sample size.

Analysis	Criteria	Minimum detectable effect/power
Bilateral correlation	n = 125; α = 0,05; power = 0,80	|rho|≈ 0,248
Regression with 3 predictors	n = 125; α = 0,05; power = 0,80	R ^2^ ≈ 0,083 (f ^2^ ≈ 0,090)
Observed model	R ^2^ = 0,067; 3 predictors	Approximate power ≈ 0,70

### Reliability of the instruments

The results of the reliability analysis using Cronbach’s alpha coefficient showed appropriate values for both variables. The variable *Strategic Communication* obtained α = 0.935, and the variable *Environmental Perception* obtained α = 0.856. These values confirm the internal consistency and reliability of the instruments used.

### Normality tests

According to the Kolmogorov–Smirnov test, none of the variables follow a normal distribution (p < 0.05), with values of 0.005 for *Strategic Communication* and 0.000 for *Environmental Perception.* For this reason, non-parametric tests were used in the correlational analysis.

## Discussion - Conclusions - Recommendations

The main finding was a virtually nonexistent overall association between perceived communication strategies and environmental perception. The width of the confidence interval excludes large associations but remains compatible with small relationships in both directions. This result partially coincides with studies that describe a poor assessment of mining communication without demonstrating that this assessment alone determines environmental attitudes. Flores Castro (2021) and Flores-Castro et al. (2022), in the context of Tía María, highlighted the perceived weakness of communication; the present study adds that a low communication assessment can coexist with a high environmental perception and that both scores do not necessarily vary together.

The absence of an overall correlation does not contradict strategic communication theory or the social license to operate. Hallahan et al. (2007) conceive of strategy as a broad organizational process, while the instrument was limited to clarity, channels, and feedback. The social license includes legitimacy, credibility, and trust ( Thomson & Boutilier, 2011), dimensions that were not measured. Therefore, a coefficient close to zero may reflect an incomplete delimitation of the construct and not the irrelevance of the company-community relationship.

Risk perception theory also helps to clarify the scope. Slovic (1987) argues that judgments about hazards incorporate control, fear, uncertainty, and trust. However, the environmental perception questionnaire assessed knowledge, attitudes, and responsibility, not mining risk. Consequently, it is not appropriate to conclude that the participants’ perceptions stem from past experiences, cultural values, or deeply held beliefs: these variables were not observed. It can only be hypothesized, for future research, that perceived risk, institutional trust, and procedural fairness could explain additional variance.

Multiple regression showed a conditional positive association for clarity, although the overall robust model was borderline and explained 6.7% of the variance. The shift from a weak bivariate coefficient to a higher adjusted one can occur when the predictors share considerable variance. In this case, clarity, channels, and feedback are part of the same scale and showed moderate collinearity. The result suggests that clarity deserves further examination, but it does not authorize its isolation as an effective intervention without replication, factor analysis, and independent measurements of trust, participation, and environmental performance.

From a social license perspective, the central finding can be interpreted as a warning against communicationism: increasing the quantity of messages does not guarantee legitimacy. The Peruvian evidence from Saenz (2019) highlights the need for consistent behaviors that build legitimacy and trust. Similarly, the IDB analysis indicates that conflict is related to territorial dissatisfaction and access to services ( Bustamante Suárez, 2024). Therefore, communication strategies should be integrated with mechanisms for dialogue, participatory monitoring, verifiable responses, and fulfillment of commitments, without assuming that communication replaces substantive decisions.

The high proportion of positive environmental perception warrants a cautious interpretation. It may indicate sensitivity and appreciation for environmental stewardship, but the knowledge subscale showed low internal consistency. This reduces the accuracy of the cognitive component and suggests revising the wording or dimensionality of the items. Responsibility and attitudes showed better reliability. Before comparing groups or making cross-cultural inferences, the instrument should be validated in Spanish and Inkawasi-Cañaris Quechua through translation procedures, cognitive interviews, and factor analysis.

### Limitations

• The actual recruitment process was not documented as random; there is a risk of selection and self-selection bias.
• The cross-sectional design prevents establishing temporality and causality.
• Demographic, language, occupation, and residence variables were not collected or incorporated into the databases; it was not possible to describe representativeness or control for sociodemographic confounding.
• The databases do not contain the literal text of the items or the Quechua version. A complete external evaluation of the content and linguistic equivalence remains pending.
• The environmental knowledge subscale showed low internal consistency.
• The communication dimensions show a high correlation, making it difficult to separate their contributions in a model with 125 observations.
• The sensitivity analysis indicates a limited capacity to detect small effects and an approximate power of 0.70 for the observed R ^2^.
• The questionnaires are self-reported and may be affected by social desirability, differential understanding, or the context of application.
• Social license, institutional trust, perceived environmental risk, prior experiences, and cultural values were not measured. None of these variables can be used as an empirical explanation of the results.

### Recommendations

1. Among the 125 participants, the overall communication strategy score did not show a statistically significant monotonic association with environmental perception (rho = 0.027; p = 0.765; 95% bootstrap CI: -0.161 to 0.207). This result means that the study found no evidence of an association, not that it proved its absence.
2. Perceptions of communication strategies were predominantly low or medium, while environmental perception was mainly high. Both patterns coexisted without appreciable overall covariation within the sample.
3. Bivariate correlations between clarity, channels, and feedback with environmental perception were small and not significant. In the robust multiple model, clarity showed a conditional positive association, but the overall evidence was borderline, the variance explained was low, and collinearity between dimensions warrants caution.
4. The available evidence does not allow us to attribute environmental perception to experiences, cultural values, beliefs, or mistrust, because these factors were not measured. Nor does it allow for establishing causality due to the cross-sectional and non-probabilistic nature of the study.
5. A more conclusive scientific evaluation requires a comprehensive and cross-culturally validated instrument, records of sociodemographic characteristics, probabilistic sampling or documented quotas, and models that incorporate trust, social license, perceived risk, and community participation.

### Implications and Recommendations

• Reformulate communication as a bidirectional and verifiable process, with clear information, documented responses, follow-up on commitments, and feedback to the community.
• Do not use communication as a substitute for environmental performance, participation, or accountability. Integrate participatory environmental monitoring and dialogue spaces with real capacity for response.
• Review the environmental knowledge items in detail and conduct cognitive interviews in Spanish and Inkawasi-Cañaris Quechua.
• Incorporate age, sex, education, occupation, mother tongue, length of residence, exposure to the project, institutional trust, perceived risk, and participation into a replication study, using categories that protect anonymity.
• Implement a longitudinal or mixed-methods design that allows for relating changes in communication, trust, and perception over time, complementing the scales with interviews or focus groups.
• Update the Zenodo repository with unambiguous files, complete instruments, consent forms, syntax or analysis script, a dictionary of variables, and a note identifying the version of each file.

## Ethics and consent

The study included 125 participants from the given population. Participation began with an in-person survey, which requested their informed consent, which was received in writing via the same means. Participants were also provided with detailed explanations about the study, including its objectives and procedures.

The research was conducted in accordance with the ethical principles established in the Declaration of Helsinki, which protects individuals involved in research. Each participant was given clear instructions and explanations about the study’s objectives, and all were offered the option to withdraw from participation at any time without repercussions. Personal information was kept confidential during and after the completion of the research.

All participants received informed consent before the start of their participation. Each participant was provided with a document detailing the study’s objectives, procedures, benefits, risks, and potential confidentiality concerns. They signed this document before completing the questionnaire. The study was conducted in compliance with the approval granted by the Institutional Ethics Committee of César Vallejo University, under code 659-2024, and adheres to the ethical principles of the Declaration of Helsinki and relevant national legislation.

The ethics committee did not grant any exemptions for the consent process, as the study methodology involved data collection through a questionnaire. It was essential that all participants provide explicit and formal consent.

Furthermore, participants were assured of confidentiality that protected their identities and provided them with the right to withdraw their participation at any time without facing negative consequences. Risks associated with personal and sensitive data were mitigated through the use of coded responses.

## Reporting guidelines

Zenodo. STROBE checklist for Communication strategies of the Cañariaco project and the environmental perception of citizens in a district of the Ferreñafe province, Perú https://doi.org/10.5281/zenodo.17315826 ( Arcila et al., 2025).

The data are available under the terms of the Creative Commons Attribution 4.0 International license (CC-BY 4.0).

## Data availability statement

### Underlying data

Zenodo: Communication strategies of the Cañariaco project and the environmental perception of citizens in a district of the Ferreñafe province, Perú https://doi.org/10.5281/zenodo.17315826 ( Arcila et al., 2025).

This article contains the following underlying data:

• Communication strategies.xlsx
• Environmental perception.xlsx
• RESEARCH METHODOLOGY.xlsx

The data are available under the terms of the Creative Commons Attribution 4.0 International license (CC-BY 4.0).

## Declarations

Authorship contributions. The CRediT contributions listed in the original version are retained: conceptualization, formal analysis, research, methodology, project management, resources, supervision, validation, visualization, data curation, and manuscript review, as applicable to each author.

Reporting guidelines. It is recommended to update the STROBE list to reflect the actual sampling, available variables, robust inference, confidence intervals, and limitations.

## Extended data

Zenodo: Communication strategies of the Cañariaco project and the environmental perception of citizens in a district of the Ferreñafe province, Perú https://doi.org/10.5281/zenodo.17315826 ( Arcila et al., 2025).

This project includes the following extended data:

• Informed consent

The data are available under the terms of the Creative Commons Attribution 4.0 International license (CC-BY 4.0).
