# Supplementary material for: Communication strategies of the Cañariaco Project and the Environmental Perception of Citizens in a District of the Ferreñafe Province, Perú
Source: F1000Res. 2026 Aug 8;14:1348. Originally published 2025 Dec 1. [Version 2] doi: 10.12688/f1000research.167797.2 (PMC13562961; doi:10.12688/f1000research.167797.2)
Supplement: Peer review history

# Reviewer report: Reviewer response for version 2

**DOI:** 10.5256/f1000research.207012.r510050

**Published:** 2026-09-01

**Authors:** MARIA DEL PILAR FERNANDEZ CELIS

**Affiliations:** 1 Universidad Nacional Pedro Ruiz Gallo, Lambayeque, Lambayeque, Peru

This second version of the manuscript is recommended for indexing because the authors have thoroughly addressed previous peer review concerns regarding theoretical grounding, corporate context, and methodological transparency. Investigating the relationship between communication strategies and environmental perception regarding the Cañariaco project ($N = 125$), the paper shows high analytical rigor by utilizing Spearman rank correlation with bootstrap confidence intervals, multiple linear regression with robust HC3 errors, and statistical sensitivity analysis. While the overall bivariate correlation between communication strategies and environmental perception is practically null and not statistically significant ($\rho = 0.027$, $p = 0.765$), the multivariate model reveals a statistically significant adjusted positive association for message clarity ($b = 0.798$, $p = 0.007$). Notably, the authors demonstrate exemplary academic integrity by explicitly addressing limitations such as nonprobabilistic sampling and omitted demographic variables while refraining from causal overstatements. This study offers valuable empirical insights and meets all scientific standards for indexing.

Is the work clearly and accurately presented and does it cite the current literature?

Yes

If applicable, is the statistical analysis and its interpretation appropriate?

Yes

Are all the source data underlying the results available to ensure full reproducibility?

Yes

Is the study design appropriate and is the work technically sound?

Yes

Are the conclusions drawn adequately supported by the results?

Yes

Are sufficient details of methods and analysis provided to allow replication by others?

Yes

Reviewer Expertise:

Social sciences

I confirm that I have read this submission and believe that I have an appropriate level of expertise to confirm that it is of an acceptable scientific standard.

# Reviewer report: Reviewer response for version 2

**DOI:** 10.5256/f1000research.207012.r509056

**Published:** 2026-08-10

**Authors:** Juan Diego Dávila Cisneros

**Affiliations:** 1 Universidad Nacional Pedro Ruiz Gallo, Lambayeque, Peru

I have nothing further to add.

Is the work clearly and accurately presented and does it cite the current literature?

Partly

If applicable, is the statistical analysis and its interpretation appropriate?

Partly

Are all the source data underlying the results available to ensure full reproducibility?

Partly

Is the study design appropriate and is the work technically sound?

Partly

Are the conclusions drawn adequately supported by the results?

Partly

Are sufficient details of methods and analysis provided to allow replication by others?

No

Reviewer Expertise:

Methodology and Data AnalysisSustainability and the Circular EconomySociology and Education

I confirm that I have read this submission and believe that I have an appropriate level of expertise to confirm that it is of an acceptable scientific standard.

# Reviewer report: Reviewer response for version 1

**DOI:** 10.5256/f1000research.184940.r463632

**Published:** 2026-05-29

**Authors:** Victor Hugo Puican Rodríguez

**Affiliations:** 1 Universidad Nacional Intercultural Fabiola Salazar Leguia de Bagua, Bagua, Peru

The manuscript addresses a timely and socially relevant topic: the communication strategies of the Cañariaco project and public perceptions of the environment. The study is timely in the Peruvian context of socio-environmental conflict linked to mining projects. We also appreciate that the authors have reported on the use of research tools, reliability (as measured by Cronbach’s alpha), correlational analysis, and access to the data on Zenodo. However, the manuscript contains significant conceptual, methodological, statistical, and scientific writing errors that undermine the robustness of its conclusions.

 In its current state, a substantial revision is necessary before it can be considered a scientifically sound contribution to a high-impact international journal. The abstract states that the study contributes to SDG 17, while the introduction mentions that it relates to SDG 16. This inconsistency must be corrected, as it undermines the manuscript’s argumentative coherence. The authors should specify which SDG the study contributes to and maintain that choice throughout the article. The theoretical framework is based on relevant background literature, but it is insufficient to address phenomena as complex as communication in the mining sector, environmental perception, and community trust. The definition of “communication strategies” draws on authors in linguistics and general communication, but does not delve into perspectives on organizational strategic communication, social license to operate, perception of environmental risk, institutional trust, or management of socio-environmental conflicts.

 We suggest incorporating specific literature on mining, community communication, risk perception, and socio-environmental conflicts in Peru and Latin America. The text refers to the Cañariaco project, but neither the operating company, nor the current status of the project, nor the history of conflict, nor the sociocultural context of the Cañaris district are adequately described. This omission undermines the interpretation of the results, since environmental perceptions cannot be understood without the territorial, historical, and community context. The manuscript describes the use of paper surveys, electronic forms, Zoom, Google Forms, and consent via email. These descriptions are contradictory and need to be clarified. It should specify whether the study was conducted in person, online, or as a hybrid; how participants were recruited; how consent was obtained; and what procedures were used to ensure understanding, voluntariness, and confidentiality.

 Although the study mentions that the population consisted of 183 citizens and the sample of 125 participants, it does not provide the sample calculation formula, confidence level, margin of error, expected proportion, or specific selection procedure. Furthermore, basic sociodemographic data on the subjects—such as age, sex, educational level, occupation, native language, and length of residence—are not reported. This information is necessary to assess the representativeness and reproducibility of the study.The manuscript excludes literate and able-bodied individuals who are unable to complete the questionnaire.

 This decision may introduce selection bias, particularly in a rural community or one where there may be Quechua speakers. The authors should either justify this exclusion or, preferably, explain what reasonable accommodations could have been made to ensure inclusion and accessibility and to meet ethical standards.The study reports the use of two expert-validated questionnaires with Cronbach’s alphas of 0.935 and 0.856, respectively. However, the items, the complete dimensions with scoring instructions, interpretation criteria, and cutoff points for categorizing low, medium, and high levels are not provided.

 This omission makes it impossible to replicate the study and assess the validity of the instrument. The results show no statistically significant relationship between communication strategies and environmental perception. However, the conclusions state that environmental perception is primarily based on past experiences, cultural values, and deeply held beliefs. This statement is not empirically supported because those variables were not measured. The conclusion should be rewritten to state that the study found no statistical evidence of a significant association, without mentioning alternative causes that were not assessed. Combining the discussion, conclusions, and recommendations into a single section makes the manuscript less clear. The discussion should compare the results with those of previous studies, discuss plausible interpretations, acknowledge limitations, and avoid making causal claims. The conclusions should be presented separately and be a direct consequence of the results.

 Spearman's correlation is appropriate for non-normally distributed data; however, bivariate analysis is not sufficient when analyzing a complex social phenomenon. It would be advisable to consider multivariate analyses or, at the very least, to include certain relevant sociodemographic variables as covariates. Effect sizes, confidence intervals, and a justification of statistical power should also be reported.

 The manuscript also lacks the descriptive statistics necessary to evaluate the magnitude and precision of the results. Although significance (p-value) values are presented, effect sizes and confidence intervals—which are crucial components in modern scientific reports of high significance because they help determine practical significance rather than merely statistical significance—are not provided. The absence of these measures makes it impossible to assess whether the lack of correlation found was due to a true lack of association between variables or to a weak statistical effect related to the sample size. A power justification (statistical power analysis) that would allow determining whether the sample has sufficient sensitivity to capture statistically significant relationships among the variables under study is also missing. This aspect is of particular importance, as an inadequate sample size can increase the risk of a type II error—that is, wrongly concluding that no relationship exists between variables when in fact there may be one. Therefore, a substantial strengthening of the statistical section is recommended, including multivariate analyses, control for relevant sociodemographic variables, estimation of effect size, confidence intervals, and statistical power analysis to provide greater methodological consistency, analytical breadth, and scientific credibility to the presented results.

Is the work clearly and accurately presented and does it cite the current literature?

Partly

If applicable, is the statistical analysis and its interpretation appropriate?

Partly

Are all the source data underlying the results available to ensure full reproducibility?

Partly

Is the study design appropriate and is the work technically sound?

Partly

Are the conclusions drawn adequately supported by the results?

Partly

Are sufficient details of methods and analysis provided to allow replication by others?

Partly

Reviewer Expertise:

NA

I confirm that I have read this submission and believe that I have an appropriate level of expertise to state that I do not consider it to be of an acceptable scientific standard, for reasons outlined above.

# Reviewer report: Reviewer response for version 1

**DOI:** 10.5256/f1000research.184940.r476986

**Published:** 2026-05-04

**Authors:** Juan Diego Dávila Cisneros

**Affiliations:** 1 Universidad Nacional Pedro Ruiz Gallo, Lambayeque, Peru

**1.    Is the work clearly and accurately presented and does it cite the current literature?**

 Partly. 

 The literature review is superficial, relies too heavily on methodology handbooks, and lacks fundamental theoretical frameworks such as social license to operate (Thomson & Boutilier), perceptions of environmental risk (Slovic), and specialized Peruvian literature on mining conflicts. Furthermore, the definition of “communication strategies” is drawn from applied linguistics (Færch, 1984) rather than organizational strategic communication.

**2.    Is the study design appropriate and is the work technically sound?**

 Partly. 

 The correlational design is consistent with the objective, but there are serious inconsistencies regarding the data collection method (in-person surveys vs. Google Forms vs. Zoom), and the exclusion criteria (illiterate individuals and people with disabilities) introduce selection bias in a Quechua-speaking community.

**3.    Are sufficient details of methods and analysis provided to allow replication by others?**

 No. 

 The questionnaire items are not presented, the sampling formula is not specified, the cutoff points for the low/medium/high categories are not defined, and the demographic characteristics of the sample are not reported.

**4.    If applicable, is the statistical analysis and its interpretation appropriate?**

 Partly. 

 The choice of Spearman's correlation is appropriate, but there is a serious error (a p-value of 4.68 cited in the background section); effect sizes and power analyses are not reported, and the bivariate analysis is insufficient for the research question.

**5.    Are all the source data underlying the results available to ensure full reproducibility?**

 Partly. 

 The data has been deposited on Zenodo (which is commendable), but there is no codebook explaining the variables.

**6.    Are the conclusions drawn adequately supported by the results?**

 Partly. 

 The classic mistake of affirming the null hypothesis is made: it is concluded that perception is based on “past experiences and cultural values” without having measured any of those variables.

 Recommended status: Not Approved, The article presents fundamental methodological and conceptual deficiencies that seriously undermine the validity of the findings and conclusions.

**REQUIRED REVISIONS FOR SCIENTIFIC SOUNDNESS**

**MANDATORY REVISIONS (must be addressed for the article to be scientifically sound)**

 M1. Identify the mining company operating the Cañariaco project and clarify the corporate context during the study period.

 M2. Resolve all contradictions regarding the data collection method (physical vs. digital surveys, in-person vs. Zoom).

 M3. Present the survey instruments in full (as appendices or supplementary material), including all items, scoring criteria, and cut-off points for the low/medium/high categorization.

 M4. Provide the sampling formula and parameters used to determine the sample size of 125 from a population of 183.

 M5. Report demographic characteristics of the sample (age, sex, education, language, occupation, residence duration).

 M6. Address the language of instrument administration (Spanish vs. Quechua) and its implications for informed consent and response validity.

 M7. Correct the overinterpretation of null findings: rewrite conclusions to reflect that absence of significant correlation does not prove absence of relationship, and that unmeasured variables cannot be invoked as explanations.

 M8. Correct the erroneous citation of 'p-value (4.68)' attributed to Hipolito et al. (2022).

 M9. Separate the Discussion from the Conclusions and provide genuine engagement with prior literature.

 M10. Redefine the construct of 'communication strategies' using organizational/strategic communication theory rather than second-language acquisition references.

**RECOMMENDED IMPROVEMENTS (strongly suggested but not strictly mandatory)**

 R1. Expand the literature review to include social license to operate (SLO) frameworks, environmental risk perception theory, and Peruvian mining conflict scholarship.

 R2. Include a power analysis to determine whether the sample size was adequate to detect meaningful effect sizes.

 R3. Conduct multivariate analyses controlling for potential confounders.

 R4. Include a codebook with the Zenodo data deposit explaining all variables, codes, and scoring procedures.

 R5. Revise the informal register of the introduction to maintain consistent academic prose throughout.

 R6. Resolve the SDG inconsistency between the abstract (SDG 17) and the introduction (SDG 16).

 R7. Include a data availability codebook alongside the deposited Excel files in Zenodo.

**REVIEWER DECLARATION**

 Competing interests: No competing interests were disclosed.

 Expertise confirmation: I confirm that I have read this submission and believe that I have an appropriate level of expertise to state that it does not meet an acceptable scientific standard in its current form, for reasons outlined above.

Is the work clearly and accurately presented and does it cite the current literature?

Partly

If applicable, is the statistical analysis and its interpretation appropriate?

Partly

Are all the source data underlying the results available to ensure full reproducibility?

Partly

Is the study design appropriate and is the work technically sound?

Partly

Are the conclusions drawn adequately supported by the results?

Partly

Are sufficient details of methods and analysis provided to allow replication by others?

No

Reviewer Expertise:

Methodology and Data AnalysisSustainability and the Circular EconomySociology and Education

I confirm that I have read this submission and believe that I have an appropriate level of expertise to state that I do not consider it to be of an acceptable scientific standard, for reasons outlined above.
